# John Home

**Published:** 1838-04

**Authors:** 


DR. JOHN HOME.

On the 18th March, at Edinburgh, Dr. John Home, son of Professor Home, a meritorious and very promising young physician. At p. 601 of the present Number we have briefly noticed the first-fruits of the zeal and industry of Dr. Home, which promised a future harvest of abundance,—alas, never to be realized!—Dr. Home fell a victim to typhus fever, caught in the discharge of his duties as physician to the Fever Hospital. It is melancholy to reflect how many of the rising lights of our profession have been extinguished by this fell scourge of the poor,—in Scotland and Ireland more especially.

